# Ethnobotanical Survey of Medicinal Plants Used by Traditional Healers to Treat Diabetes in the Taza Region of Morocco

**DOI:** 10.1155/2021/5515634

**Published:** 2021-04-24

**Authors:** Hanae Naceiri Mrabti, Abdelhakim Bouyahya, Nidal Naceiri Mrabti, Nidal Jaradat, Latifa Doudach, My El Abbes Faouzi

**Affiliations:** ^1^Laboratory of Pharmacology and Toxicology, Bio Pharmaceutical and Toxicological Analysis Research Team, Faculty of Medicine and Pharmacy, Mohammed V University in Rabat, BP 6203, Rabat, Morocco; ^2^Laboratory of Human Pathologies Biology, Department of Biology, Faculty of Sciences, and Genomic Center of Human Pathologies, Mohammed V University in Rabat, Rabat, Morocco; ^3^Computer Chemistry and Modeling Team, Laboratory of Materials, Modeling and Environmental Engineering, LIMME, Faculty of Sciences Dhar El Mehraz, Sidi Mohammed Ben Abdellah University, USMBA, BP.1796, 30000, Atlas, Fez, Morocco; ^4^Department of Pharmacy, Faculty of Medicine and Health Sciences, An-Najah National University, P.O. Box 7, Nablus, State of Palestine; ^5^Department of Biomedical Engineering Medical, National School of Arts and Crafts Rabat (ENSAM), Mohammed V University, BP 6203, Rabat, Morocco

## Abstract

Type 2 diabetes is one of the noncommunicable diseases that is becoming a pandemic in Africa. In Morocco, traditional healers have started to use herbal medicines for the treatment of diabetes either individually or in combination with food. The current study aimed to perform an ethnobiological survey of antidiabetic plants use in the Taza region of Morocco. A total of 193 traditional healers were interviewed using a semistructured questionnaire. Data collected were analyzed utilizing the use value (UV), fidelity level (FL), and relative frequency citation (RFC) indices. Forty-six plant species belonging to 28 families were recorded for the treatment of diabetes in the Taza region of Morocco. The most frequently cited plant species are *Salvia officinalis*, *Marrubium vulgare*, and *Ajuga iva.* Lamiaceae, Asteraceae, and Fabaceae were the most reported families. Leaves are the most used part of plants to prepare drugs, the decoction is the preferred mode of preparation, and remedies are often administered orally. Interestingly, *Cytisus battandieri, Urginea maritima, Plantago ovata,* and *Ziziphus jujuba* were reported as new medicinal plants used to treat diabetes in the Taza region of Morocco. People in the Taza region still rely on indigenous plants for their basic healthcare needs. Further research should be carried out to validate the antidiabetic effect of the newly reported plant species. This validation can be investigated by the determination of bioactive compounds and evaluation of their *in vitro* and *in vivo* antidiabetic effects.

## 1. Introduction

Herbal medicine is an alternative or a parallel treatment in many acute and chronic diseases. It is generating renewed interest in many countries around the world, particularly in North Africa. Indeed, a large number of plants are used in traditional medicine in Morocco, including some for the treatment of diabetes [1]. Despite the presence of antidiabetic drugs known on the pharmaceutical market, herbal remedies are importantly used to treat this disease [2]. Indeed, good knowledge and authentication of medicinal species in particularly by their vernacular names can avoid the problems of their side effects and toxicities [3]. However, for millennia, herbal medicines have been a valuable source of therapeutic agents, and many of today's drugs are inspired from natural herbal plants or their derivatives [4].

Scientific research on traditional herbal remedies for diabetes can provide valuable leads for the development of alternative medicines and strategies [5, 6]. Despite numerous studies carried out in Morocco to bring up rational science about the use of plants by the local populations [1, 7–9], there is still a need to pursue investigation in order to have a comprehensive database on medicinal plants used for diabetes management. The available literature showed several Moroccan medicinal species with antidiabetic properties [6]. Although some of these plants have a great reputation in traditional medicine, many remain to be scientifically demonstrated [10].

In Morocco, as in developing countries, the use of traditional medicine is widespread, and several herbal remedies used individually or in combination with conventional medicines are recommended to treat diabetes [8]. Indeed, recently an ethnobotanical survey was carried out in the Beni Mellal region of Morocco [11] to collect the data about the traditional herbal medicine used for diabetes treatment in this region. So far, no scientific investigation has been carried out in the Taza region. Therefore, the current study aims to perform an ethnopharmacological survey on traditional knowledge of medicinal plants used to treat diabetes in the Taza region.

## 2. Materials and Methods

### 2.1. Study Area

**Figure 1:**
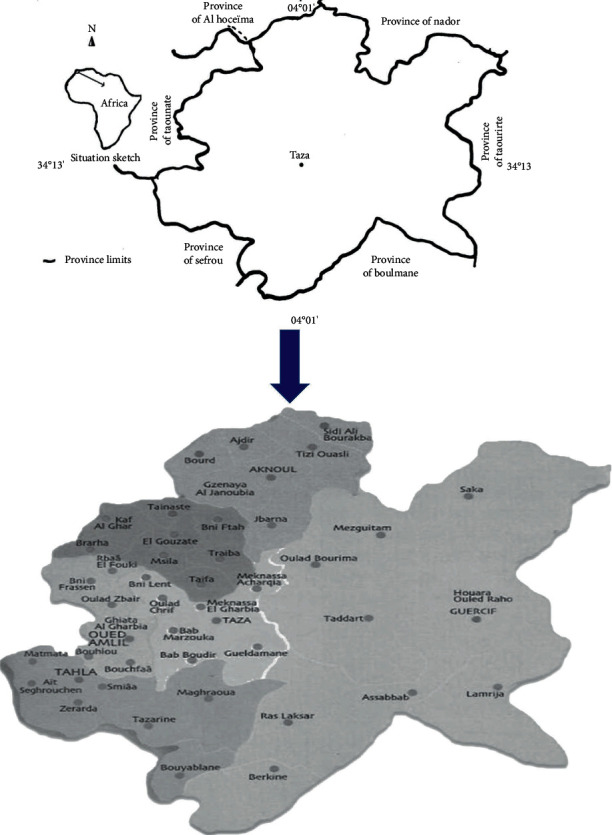
Map of the study area.

This study was conducted in the province of Taza, situated in the northeast of Morocco. It is bounded by the Rif in the North, the Middle Atlas in the South, the Rharb plain in the West, and the Guercif plain in the East (Figure 1); it constitutes the northern termination of the Middle Atlas and extends over a length of 50 km and a width of 15 km. The altitudes are between 500 and 1980 m. The temperature varies between 3.2 and 44.5°C. Although annual rainfall in this region has ranged between 84 and 120 mm over the last 8 years [12], the climate is subtropical to tropical, very dry on the coast, and wetter at altitude.

### 2.2. Ethnobotanical Survey

An ethnobotanical survey was conducted from March 2019 to February 2020, and before starting the collection of ethnobotanical data, a brief explanation to the informants on the objectives of the study and the importance of the information they would provide was provided in order to obtain their consent to participate in the study. A total of 193 healers were interviewed for this purpose. The data were collected through semistructured interviews using Moroccan language (Darija). These interviews were designed to record information about the plants used to treat diabetes mellitus and their local names, the used parts of the plant, the methods of preparation, the administration of drugs, and the demographic characteristics of the study participants.

### 2.3. Preservation of Plant Specimens

The standard method was followed with a record to collect the plant materials, drying, mounting, preparation, and preservation in special glass frames; they were later identified by Pr. Latifa Doudach. These samples of plant material were given herbarium specimen codes, and the voucher plant samples were kept in the Herbarium of the Botany Department of the Scientific Institute of Rabat, Morocco. The complete floristic list was established after the identification and verification of the samples; the identification process included the Traditional Moroccan Pharmacopeia [13], Practical Flora of Morocco [14], Moroccan Medicinal and Aromatic Plants [15], and Vascular Flora of Morocco [14] as references. The taxonomy was confirmed based on data available on the International Plant Names Index website: http://www.ipni.org/.

### 2.4. Data Analysis

Medicinal plants inventoried in this study were organized in alphabetical order by family. The data reported concerned family, scientific name, local name, part used the utilized preparations, main therapeutic use, and identification code. The obtained results were analyzed using specific quantitative parameters.

#### 2.4.1. Use Value (UV)

The use value (UV) demonstrates the relative importance of plants known locally in traditional medicine [16]. Use value (UV) was calculated using the following formula:

(1) $$\begin{array}{c} \text{UV}=\frac{\sum U}{n}, \end{array}$$

where UV is the use value of species, *U* is the number of respondents who mentioned the use of the species, and *n* is the total number of respondents interviewed for a given species (herbalists and local population). The highest use recorded in inquiry reports for a given plant gives a rise to the highest UV value.

#### 2.4.2. Fidelity Level (FL)

Fidelity level (FL) indicates the percentage of informants claiming the use of a certain plant species for the same major purpose [17]. The fidelity level is calculated by the following formula:

(2) $$\begin{array}{c} \text{FL }\left(\%\right)=\frac{N_{p}}{N}\times 100, \end{array}$$

where *N*_*p*_ is the number of informants who independently indicated the use of a species for the same major ailment and *N* is the total number of informants who mentioned the plant for any major ailment.

#### 2.4.3. Relative Frequency Citation (RFC)

The collected ethnomedicinal information was quantitatively analyzed using an index of relative frequency citation (RFC) (0 < RFC < 1):

(3) $$\begin{array}{c} \text{RFC}=\frac{\text{FC}}{N}. \end{array}$$

This index shows the local importance of each species, and it is given by the frequency of citation (FC, the number of informants mentioning the use of the species) divided by the total number of informants participating in the survey (*N*), without considering the use categories [16].

## 3. Results and Discussion

### 3.1. Demographic Features

This study enrolled 193 informants, including traditional healers, herbalists, and knowledgeable villagers represented by 169 women (87.56%) and 24 men (12.44%), and they were interviewed (Table 1). The results showed that women were more involved in the use of medicinal plants than men, and this could be explained by the activities of women at home. Indeed, they were responsible for drying, storing, and the preparation of the herbal remedy for maintaining the healthcare of their family. Their average age was 59.5 years with a minimum of 32 years and a maximum of 87 years. The age group [18–38] was the most represented with 46.11%. The majority of the respondents belong to the rural area (92%); and 84.45% are illiterate, 12.95% primary education, 2.07% secondary education, and 0.51% university education. Regarding the origin of their knowledge, the majority of them (96.89%) who participated in this survey acquired the traditional medical knowledge of their family members, mainly grandparents and parents, and this knowledge has not been documented.

### 3.2. Medicinal Plants and Floristic Analysis

This ethnobotanical survey revealed the presence of 46 plant species, belonging to 28 families used for the treatment of diabetes by participants from the Taza region, and these species were reported along with their medicinal uses and their methods of use (Table 2). The most common medicinal plant family is Lamiaceae (6 species), Asteraceae and Fabaceae (5 species each), and Apiaceae and Myrtaceae (3 species each), respectively, whereas the other families were represented with a variable number of fewer than 2 species.

### 3.3. Ethnobotanical Indices

In this study, the analysis of data collected in the field allowed us to determine forty-six (46) most commonly cited plants known to most medical informants about the treatment of diabetes disease (Table 2). The obtained data were analyzed and ordered based on the value of use (UV), level of accuracy (FL), and relative citation frequency (RFC). The values of the collected plant species ranged from 0.01 to 1.63 for UV; 8.33 to 95.34% for FL, and 0.01 to 1 for RFC, respectively. Among 46 inventoried species, ten plants species identified had high FL values (FL of ˃50%), which are *Salvia officinalis*, *Marrubium vulgare, Trigonella foenum-graecum*, *Olea europaea*, *Artemisia herba-alba*, *Ajuga iva, Arbutus unedo, Euphorbia resinifera, Taraxacum officinale,* and *Ceratonia siliqua.* Likewise, while *Salvia officinalis* (1.00) has the highest value of RFC followed by *Marrubium vulgare* (0.97) and *Ajuga iva* (0.94), respectively. These positions correspond to the fact that these plants were reported by the highest number of informants and RFC directly depends on the number of informants mentioning the use of this plant (FC). As shown in Table 2, *S. officinalis* has the highest use value (1.63) followed by *M. vulgare* (1.53) and *A. iva* (1.46).

### 3.4. Comparative Analysis with the Ethnobotanical Literature

Four medicinal plants have been mentioned for the first time to treat diabetes. Indeed, the literature reviewed showed that these plants have not been reported by other Moroccan studies. These species include *Cytisus battandieri, Urginea maritima, Plantago ovata,* and *Ziziphus jujuba* (Table 2).

### 3.5. Plant Parts Used, Mode of Preparation, and Administration

**Figure 2:**
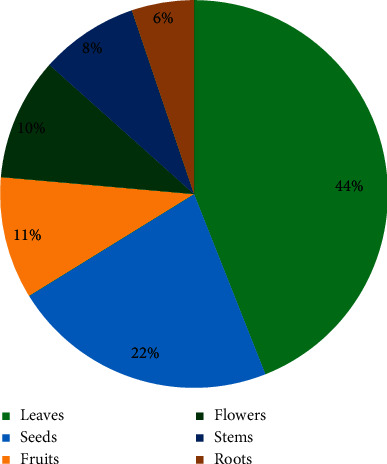
Medicinal use of medicinal plants of the Taza area against diabetes according to plant parts used.

The data showed that the leaves were the most used part (44%) of the medicinal plants followed by seeds (22%), flowers and fruits (10% each), stems (8%), and roots (5%) (Figure 2).

**Figure 3:**
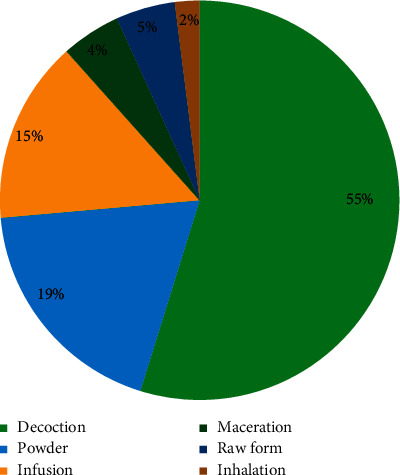
Medicinal use of medicinal plants of the Taza area against diabetes according to the preparation methods.

The decoction was the primary mode of preparation of herbal medicines accounting for 55% followed by powder (19%), infusion (15%), raw form (5%), maceration (5%), and inhalation (2%) (Figure 3).

### 3.6. Antidiabetic Effects of Medicinal Plants Cited in This Survey

*In vitro* and *in vivo* antidiabetic effects of some of the cited plants in this survey were investigated. Indeed, four medicinal plants (*A. iva, Arbutus unedo*, *Calendula arvensis*, and *Ziziphus lotus*) were tested *in vitro.* Twelve plants (*A. iva, Calamintha officinalis*, *Carum carvi*, *Ammi visnaga*, *Lepidium sativum*, *Chamaerops humilis*, *Capparis spinosa*, *Arbutus unedo*, *Eucalyptus globulus*, *Nigella sativa,* and *Urtica dioica*) were tested *in vivo* (Table 3).

## 4. Discussion

This study reports the medicinal plants used in the Taza region to treat diabetes. The demographic features showed that women were often at home during survey hours. Some previous studies have also shown this trend [7, 8, 11]. Moreover, the interviews showed that the elderly were particularly competent and had better knowledge of plants than the young; similar results have been observed in other studies where indigenous knowledge on the use of medicinal plants is still strong with the elderly [81–83]. On the other hand, results revealed that the majority of the respondents belong to rural areas and are illiterate. These results are corroborated by other studies, which have shown that illiterate people have more expertise in the uses of traditional medicine [5].

Medicinal plants diversity showed a floristic diversity. Indeed, the findings were in agreement with previous studies, where these families were the most represented in diabetes mellitus treatment in Morocco [8, 44, 47, 64], Turkey [84], and Togo [85]. The reasons for the high degree of ethnomedicinal plants of families Lamiaceae, Asteraceae, Fabaceae, Apiaceae, and Myrtaceae in the region is their wide occurrence with several traditional uses known by the local informants [8].

The survey revealed that *S. officinalis, M. vulgare,* and *A. iva* are the most used in traditional medicine to treat diabetes in this region. Literature reports showed that several of these plants have been reported in other ethnomedical surveys for the treatment of diabetes [7, 44, 45, 47]. The antidiabetic activity of certain plants has also been experimentally proven by *in vivo* or *in vitro* studies. These species include *A. herba-alba* [21, 33, 34]*, S. officinalis* [27]*, O. europaea* [29]*, A. iva* [35]*, A. unedo* [63], and *M. vulgare* [24]. Actually, certain mentioned plants open up interesting perspectives in the search for new therapeutic means, which can thus provide credible solutions by the production of low-cost and effective drugs for the treatment of diabetes.

However, the use of *C. battandieri, U. maritime, P. ovate,* and *Z. jujube* for managing diabetes has not been reported. International literature has shown *P. ovate* and *Z. jujube* have been used for diabetes in other countries like Iran [30, 31, 36]. The majority of these plants have been reported as medicinal plants in the treatment of other diseases*. C. arvensis* has been used as a disinfectant, antispasmodic, antipyretic, anti-inflammatory, antiepileptic, antimicrobial, and diuretic [37, 38]. *Urginea maritima* was used for the treatment of cardiac failure, chronic bronchitis, asthma, and diuretic [86]. *Salix alba* has been used for the treatment of various ailments due to its potent antipyretic, analgesic, and anti-inflammatory properties [87].

In addition, the part of the plant most frequently used in this study was the leaves, which corresponds to previous studies that declared that the leaves are part of the plant mainly used in the treatment of diabetes in Morocco [8, 11], Kenya [88], Togo [81], and Pakistan [23]. The reason for the high use of leaves can be explained by their availability and their ease of collection compared with other parts like roots, flowers, and fruits [89].

Concerning the mode of preparation, similar types of results were obtained in other studies, where decoction was the most frequent mode of preparation [11, 17, 25, 41]. The most frequently used mode of herbal remedy administration is oral ingestion (98.30 %). Today, most of the medicines were given orally, which is in agreement with other studies [11, 90, 91].

A bibliometric study was carried out to identify which cited medicinal plants were already tested *in vitro* and *in vivo* for their antidiabetic effects. *In vitro* investigations showed that some plants were studied *in vitro.* Indeed, Abudunia et al. [78] have tested the *in vitro* antidiabetic effects of methanolic, hexane, and aqueous extracts of *C. arvensis* collected from the region of Khemisset. All tested extracts exhibited enzymatic inhibitory effects on *α*-amylase, *α*-glucosidase, and *β*-glucosidase with some variability. Indeed, methanolic extract seems to be the most effective product, in particular, against *α*-amylase (IC_50_ = 573.37 ± 36.85 *µ*g/mL) and *α*-glucosidase (IC_50_ = 848.83 ± 49.93 *µ*g/mL). Saad et al. [35] showed that aqueous and methanolic extracts of *Ajuga iva* exhibited remarkable inhibition of *α*-amylase (IC_50_ = 0.210 ± 0.003 and IC_50_ = 0.180 ± 0.005 *µ*g/mL, respectively) and *α*-glycosidase (IC_50_ = 0.172 ± 0.012 and IC_50_ = 0.130 ± 0.008 *µ*g/mL, respectively). The authors suggested that these effects are related to the phenolic compounds present in plant extracts. Moreover, the acute toxicity evaluation on rats showed that these extracts are not toxic [35].

The root aqueous extract of *A. unedo* has revealed significant enzyme inhibition of *α*-amylase and *α*-glucosidase [63]. The inhibitory effect was significantly important against *α*-glucosidase (IC_50_ = 94.81 ± 5.99 mg/mL). This remarkable result led Naceiri Mrabti and collaborators to fractionate the root aqueous extract of *A. unedo* and to isolate the major compounds. The results revealed the presence of catechin as the main compound, which was isolated and tested for its inhibitory effect against *α*-glucosidase [92]. Indeed, the inhibitory activity of *α*-glucosidase was increased (IC_50_ = 87.55 ± 2.23 mg/mL), compared with the inhibitory activity of aqueous extract [92]. Marmouzi et al. [79] tested the methanolic extracts of leaves and fruits of *Z. lotus* (Rhamnaceae) showed significant inhibition of *α*-amylase and anti-*α*-glucosidase at lower concentrations with some differences between fruit and leaf extracts. Indeed, the leaf extract revealed higher inhibitory potency as anti*-α*-amylase and anti-*α*-glucosidase compared with the fruit extract for which IC_50_ = 20.40 ± 1.30 and IC_50_ = 8.66 ± 0.62 mg/mL, respectively [79].

However, several medicinal plants cited in this survey have shown important in vivo antidiabetic effects. *U. dioica* aqueous extracts were tested using the oral glucose tolerance test and intravenous glucose tolerance test and showed that the administration of extract orally at 250 mg/kg decreased glycemia significantly, which indicates a significant inhibition of glucose absorption. Moreover, using oral glucose tolerance on normal and streptozotocin-induced diabetic rats has shown that the same extract decreases plasma glucose levels at 27.4% [62]. Moreover, at the same concentration, another study revealed that the antidiabetic effect was also accompanied by hypolipidemic action [65]. *Calamintha officinalis* is another Moroccan antidiabetic medicinal plant that belongs to Lamiaceae family. On the other hand, Eddouks et al. [70] reported that the *L. sativum* aerial part aqueous extract (Brassicaceae) administered orally at 20 mg/kg exhibits a potent hypoglycemic effect without affecting basal plasma insulin. However, oral and intravenous administration of the same extract at 10 mg/kg/h in normal and STZ-induced diabetic rats caused a significant reduction of blood glucose levels in both normal and diabetic rats. Therefore, this extract normalized glycemia, enhanced glycosuria, and decreased the amount of urinary TGF-*β* 1 in diabetic rats. These suggest that the plant extract has a potent inhibition of renal glucose reabsorption, which reduces blood sugar [70]. Eddouks et al. [70] showed that the oral administration of *C. humilis* leaf aqueous extract at 10 mg/kg decreased plasma glucose levels in normal and obese-hyperglycemic-hyperlipidemic (OHH) Meriones shawi rats. Moreover, another study showed that, at the dose of 20 mg/kg, the hypoglycemia effect was also accompanied by the potent inhibition of renal glucose reabsorption [71]. However, *in vitro* study using short-circuit current technique revealed that the aqueous extract caused significant inhibition of the electrogenic intestinal absorption of glucose [75]. Bnouham et al. [80] studied the hypoglycemic effect of aerial part aqueous extracts of *U. dioica* (Urticaceae) using oral glucose tolerance test (OGTT) at a dose of 250 mg/kg in normal and alloxan-induced diabetic rats. The results showed a strong glucose-lowering effect in normal rats and no effect in alloxan-induced diabetic rats. In another work, intraperitoneal administration of aerial part aqueous extracts of this plant at a dose of 400 mg/L in the chronic treatment of streptozotocin-induced diabetic rats and 150 mg/kg in the oral glucose tolerance test caused a significant reduction of glycemia in oral glucose tolerance and plasma glucose levels [62].

The study highlighted results about antidiabetic medicinal plants as a source of antidiabetic drugs for further investigations, in particular for medicinal plants that have not been yet investigated. However, some limitations were found during this study. The first concerns the limits in the samples of traditional healers consulted because the majority of people with significant traditional knowledge refuse to provide information through the interviews. Indeed, some participants refuse to give the names of certain plants because they consider that it is a secret in itself and quickly it limits the transmission of this ancestral knowledge. The second limitation concerns the intellectual level of these traditional healers who risk passing information that is not clear enough. The number of traditional healers interviewed is also an important limitation of this study. This limit is indeed due to the low participation of traditional healers in this survey. Moreover, other difficulties related to reaching certain areas of this region because of a bad infrastructure, which justifies the reduced number of questionnaires. In addition, despite the efforts to reach some of these regions, the questionnaire has not been completed because these areas speak the Tamazight language, which we do not speak. In this sense, future work with a well-equipped research team full of linguistic skills could yield other interesting information about traditional knowledge.

## 5. Conclusion

This work has enabled us to inventory forty-six medicinal species used in traditional medicine in the treatment of diabetes, in the Province of Taza. The frequency of the use of medicinal plants is very linked to the profile of the interviewees; younger people generally ignore this know-how. Women and men have shared medicinal knowledge, with a slight difference in favor of the former in terms of the use of medicinal plants. The most widely used medicinal plants in the studied region belong to 28 families, the most popular of which are Lamiaceae, Asteraceae, and Fabaceae. In traditional medicine, the leaves mainly represent the parts of the plant used. The decoction is the galenic form most practiced by the local population whose oral route constitutes the most used route of administration. This study allowed us to appreciate and know the practices of traditional medicine in the treatment of diabetes, transmitted by the population in this region.

## Figures and Tables

**Table 1:** Sociodemographic characteristics and experience of herbalists.

Characteristics	Number of informants (*n*)	Frequency (%)
Ages (years)		
30–50	56	29.01
50–70	89	46.11
70–90	48	24.87
Total	**193**	**100**

Gender		
Male	24	12.44
Female	169	87.56
Total	**193**	**100**

Education		
Illiterate	163	84.45
Primary	25	12.95
Secondary	4	2.07
University	1	0.51
Total	**193**	**100**

Origin of knowledge		
Family heritage	187	96.89
Traditional initiation	6	3.11
Total	**193**	**100**

**Table 2:** Medicinal plants used to treat diabetes in the Taza region (Morocco).

Family name	Plant species	Voucher codes	Vernacular name	Parts used	Preparation	Administration	UV	FL	RFC	Recorded literature for antidiabetic uses in Morocco	Recorded literature for antidiabetic uses out Morocco
Amaranthaceae	*Chenopodium ambrosioides* L.	RAB13518	Mkhinza	Leaves	Infusion	Oral	0.20	28.57	0.07	[11, 39–42]	Nd
Anacardiaceae	*Pistacia atlantica* Desf.	RAB 1372	Drou	Fruits	Decoction	Oral	0.11	30.00	0.05	[8, 43]	Nd
Apiaceae	*Carum carvi* L.	RAB7119	Lkarwya	Seeds	Decoction Maceration Infusion Powder	Oral	0.16	41.67	0.06	[8, 11, 42, 44–46]	Nd
Apiaceae	*Foeniculum vulgare* Mill.	RAB9217	Nafaa	Seeds	Decoction	Oral	0.30	47.83	0.12	[8, 11, 40, 44–46]	Nd
Apiaceae	*Ammi visnaga* (L.) Lam	RAB12423	Bachnikha	Fruits	Decoction	Oral	0.35	35.29	0.09	[11, 13, 41, 45, 47–50]	Nd
Apocynaceae	*Nerium oleander* L.	RAB18820	Defla	Leaves	Fumigation	Inhalation	0.18	14.29	0.07	[11, 43–47, 51, 52]	nd
Asteraceae	*Artemisia herba-alba* Asso.	RAB43124	Chih	Stems Leaves Roots	Decoction Infusion	Oral	0.90	69.35	0.32	[8, 11, 13, 41, 42, 44, 45, 47–49, 52, 53]	nd
Asteraceae	*Matricaria chamomilla* L.	RAB12321	Babounj	Flowers	Decoction Infusion	Oral	0.38	29.03	0.16	[8, 11, 13, 41, 42, 44, 45, 47–49, 52, 53]	[54]
Asteraceae	*Dittrichia viscosa* (L.) Greuter	RAB14422	Magramane	Leaves Roots	Decoction	Oral	1.45	32.20	0.61	[50]	nd
Asteraceae	*Taraxacum officinale* L.	RAB14324	Garnina	Leaves Roots	Decoction Powder	Oral	0.14	57.14	0.11	[55]	[56, 57]
Asteraceae	*Calendula arvensis* L.	RAB14312	Zwiwl	Stems Flowers	Decoction	Oral	0.07	9.09	0.06	[58]	
Brassicaceae	*Lepidium sativum* L.	RAB14317	Habbrchad	Seeds	Infusion	Oral	0.62	38.33	0.31	[11, 44, 59]	[60]
Cactaceae	*Opuntia ficus-indica* (L.) Mill.	RAB12411	Lhndia	Stems Flowers	Decoction Powder	Oral	0.41	20.51	0.20	[8, 11, 44]	nd
Capparaceae	*Capparis spinosa* L.,	RAB97161	Lekbar	Fruits	Decoction	Oral	0.32	43.48	0.12	[11, 45, 49, 53]	[61]
Cupressaceae	*Tetraclinis articulata* Benth.	RAB18717	Al'Araâr	Leaves	Decoction	Oral	0.19	38.10	0.11	[7, 11, 41, 45, 49, 50, 55]	nd
Ericaceae	*Arbutus unedo* L.,	RAB 101549	Sasnou	Leaves	Decoction	Oral	0.43	59,52	0.22	[7, 11, 41, 45, 49, 50, 55, 62, 63]	nd
Euphorbiaceae	*Euphorbia resinifera* Berg.	RAB14822	Ssekoum	Leaves	Decoction	Oral	0.35	57.58	0.17	[11, 18, 50]	nd
Fabaceae	*Ceratonia siliqua* L.,	RAB38628	Lkharoub	Leaves Seeds	Decoction Powder	Oral	0.26	52.63	0.10	[11, 19, 47, 50]	nd
Fabaceae	*Glycine max* (L.) Merr.	RAB07616	Soja	Seeds	Decoction Powder	Oral	0.70	45.65	0.24	[8, 11]	nd
Fabaceae	*Trigonella foenum-graecum* L.	RAB24117	Lhelba	Seeds	Decoction Powder	Oral	1.01	72.73	0.40	[7, 11, 13, 19, 40–42, 44, 46, 49, 53, 59]	[2, 20–23]
Fabaceae	*Lupinus albus* L.	RAB21118	Termes	Seeds	Powder	Oral	0.28	41.67	0.06	[11, 18, 45, 47, 55]	nd
Fabaceae	*Cytisus battandieri* Maire	RAB23246	Akhamelel	Leaves	Decoction	Oral	0.04	33.33	0.02	Nd	nd
Gentianaceae	*Centaurium erythraea* Rafn	RAB22415	Kassatlahya	Flowers	Decoction	Oral	0.44	21.21	0.17	[13, 45, 48–50, 55]	nd
Lamiaceae	*Ajuga iva* (L.) Schreb.	RAB04	Chndkoura	Stems Leaves	Powder	Oral	1.16	65.78	0.94	[8, 11, 13, 41, 43, 46]	[60]
Lamiaceae	*Marrubium vulgare* L.	RAB47249	Mriwt	Leaves	Decoction	Oral	1.35	81.28	0.97	[8, 11, 19, 43, 44, 59]	[24, 60]
Lamiaceae	*Salvia officinalis* L.	RAB61862	Salmia	Leaves	Decoction Infusion	Oral	1.63	95.34	1.00	[8, 11, 18, 19, 42, 43, 49, 53, 64]	[25–27]
Lamiaceae	*Calamintha officinalis* Moench	RAB6922	Manta	Leaves Stems Flowers	Decoction Infusion	Oral	0.37	40.00	0.05	[11, 49, 64]	nd
Lamiaceae	*Thymus broussonetii* Boiss.	RAB40441	Zaitra	Leaves	Decoction	Oral	0.63	33.33	0.08	[8, 19]	nd
Lamiaceae	*Rosmarinus officinalis* L.	RAB8088	Azir	Leaves	Decoction	Oral	0.21	25.00	0.06	[11, 19, 44, 45, 49–51, 55]	nd
Liliaceae	*Urginea maritima* (L.) Baker	RAB23142	Bssallansal	Leaves	Decoction	Oral	0.01	16.67		Nd	nd
Moraceae	*Ficus carica* L.	RAB8217	Lkarmous/Chriha	Leaves	Decoction	Oral	0.34	16.67	0.03	[7, 8, 11, 18, 41, 44, 58]	[23]
Myristicaceae	*Myristica fragrans* Houtt.	RAB1161	Lgouza	Seeds	Powder	Oral	0.33	30.00	0.05	[8, 11]	[60]
Myrtaceae	*Eucalyptus globulus* Labill (sp.)	RAB9318	Al'Kalitouss	Leaves fruits	Decoction	Oral	0.45	33.33	0.06	[7, 11, 18, 43, 45, 49, 51, 59]	[6, 28]
Myrtaceae	*Eugenia caryophyllata* Thunb.	RAB41219	Qronfel	Leaves Flowers	Decoction Powder Maceration	Oral	0,28	20,00	0,01	[7, 11, 45, 49, 59, 64]	nd
Myrtaceae	*Myrtus communis* L.	RAB49621	Arraihan	Leaves Fruits	Decoction Infusion	Oral	0.18	12.50	0.04	[7, 11, 41, 43–45, 49, 51, 59]	[25]
Oleaceae	*Olea europea* L.	RAB51119	Zaytoun	Leaves	Decoction	Oral	1.53	77.60	0.65	[8, 11, 19, 41, 43–47, 49]	[29, 60]
Papaveraceae	*Papaver rhoeas* L.	RAB51218	Belaaman	Seeds	Powder	Oral	0.66	23.53	0.09	[8, 11]	[25]
Plantaginaceae	*Plantago ovata* Forssk.	RAB003	Katouna	Seeds	Infusion	Oral	0.06	20.83	0.12	Nd	[30]
Ranunculaceae	*Nigella sativa* L.	RAB35821	Sanûj	Seeds	Powder	Oral	1.08	37.10	0.32	[11, 19, 43, 44, 49, 59, 64]	[2, 23, 60]
Rhamnaceae	*Ziziphus lotus* (L.)	RAB62218	Nbeg	Leaves	Decoction Powder	Oral	0.50	25.00	0.02	[7, 8, 11, 41, 44, 46, 47]	[60]
Rhamnaceae	*Ziziphus jujuba* Mill		Zafzouf	Leaves	Decoction	Oral	0.61	22.22	0.28	Nd	[31]
Rosaceae	*Prunus amygdalus* Batsch	RAB55417	Louzmarr	Seeds	Raw	Oral	0.88	23.08	0.03	[7, 13, 41, 45, 47, 49, 53, 59]	[23]
Rutaceae	*Citrus aurantium* L.	RAB62131	Larnaj	Fruits	Raw	Oral	0.90	33,33	0,01	[44, 53]	[32]
Salicaceae	*Salix alba* L		Aouad lma	Leaves	Decoction	Oral	0.09	28.57	0.04	[51, 52]	nd
Urticaceae	*Urtica dioica* L.	AB56517	Lhriga	Stems Leaves	Decoction	Oral	0.25	12.50	0.04	[7, 8, 11, 41, 49, 59]	[25]
Zygophyllaceae	*Peganum harmala* L.	RAB41116	Lharmel	Seeds	Maceration	Oral	0.01	16.67	0.03	[7, 41, 45, 49, 59]	[25, 60]

**Table 3:** In vitro and in vivo antidiabetic cited medicinal plants in this survey.

Species	Part used	Extracts	Dose/rate of administration	Experimental model	Key results	References
*Ajuga iva*	Whole plant	Aqueous extract	10 mg/kg orally	Normal and STZ-induced diabetic rats	Strong hypoglycemic effect in diabetic rats significantly decreased in plasma glucose level	[46]
	Whole plant	Aqueous extract	10 mg/kg orally	Normal and STZ-induced diabetic rats	Hypolipidemic and hypoglycemic activity in diabetic rats	[65]
	Aerial parts	Aqueous extract	—	*α*-Amylase inhibition	IC_50_ = 0.210 ± 0.003 *µ*g/mL	[35]
			—	*α*-Glucosidase inhibition	IC_50_ = 0.180 ± 0.005 *µ*g/mL	
		Methanolic extract	—	*α*-Amylase inhibition	IC_50_ = 0.172 ± 0.012 *µ*g/mL	
			—	*α*-Glucosidase	IC_50_ = 0.130 ± 0.008 *µ*g/mL	

*Calamintha officinalis*	Aerial part	Aqueous extract	100 mg/kg, during 3 weeks orally	Diabetes mellitus model of the mouse (high-fat diet orally)	Antidiabetic activity, a loss of weight as well as a decrease in the free fatty acid plasmatic concentrations	[66]
	Aerial part	Aqueous extract	20 mg/kg, single or daily oral	Normal and STZ-induced diabetic rats	Significant hypoglycemic effect without affecting basal plasma insulin concentrations.	[67]

*Carum carvi*	Fruits	Aqueous extract	Single oral dose (20 mg/kg) or 14 daily doses orally	Normal and STZ-diabetic rats	Significant decreases in blood glucose levels in STZ diabetic rats without affecting basal plasma insulin concentrations but not high changes in blood glucose were observed in normal rats.	[68]

*Ammi visnaga*	Aerial part	Aqueous extract	Single and repeated dose of 20 mg/kg orally	Normal and STZ-induced diabetic rats	The extract possesses a significant hypoglycemic effect in both normal and STZ diabetic rats.	[69]

*Lepidium sativum*	Aerial part	Aqueous extract	Single dose (20 mg/kg) or chronic 15 daily repeat administration orally	Normal and STZ-induced diabetic rats	Aqueous extract of LS exhibits a potent hypoglycemic activity in rats without affecting basal plasma insulin concentrations.	[70]
	Aerial part	Aqueous extract	10 mg/kg/h intravenously and orally	Normal and STZ-induced diabetic rats	Extract reduced blood glucose levels both in normal and diabetic rats. Oral administration of LS for 15 days normalized glycemia enhanced glycosuria and decreased the amount of urinary TGF-*β* 1 in diabetic rats. The extract caused a potent inhibition of renal glucose reabsorption, which in turn reduced blood sugar.	[71]

*Chamaerops humilis*	Leaves	Aqueous extract	Single and chronic dose (10 mg/kg)	Normal and obese-hyperglycemic-hyperlipidemic (OHH) Meriones shawi rats	Plant extract decreased plasma glucose levels	[72]

*Capparis spinosa*	Fruits	Aqueous extract	20 mg/kg orally	Normal and STZ-induced diabetic rats)	Significant antihyperglycemic activity in STZ rats without affecting basal plasma insulin concentrations	[1]
	Aerial part	Aqueous extract	100 mg/kg, during 3 weeks orally	Diabetes mellitus model of the mouse (high-fat diet orally)	Antidiabetic activity, a loss of weight as well as a decrease in the free fatty acid plasmatic concentrations	[73]

*Arbutus unedo*	Roots	Aqueous extract	400 mg/L in the chronic treatment of streptozotocin-induced diabetic rats (intraperitoneally) 150 mg/kg in oral glucose tolerance test (orally)	Oral glucose tolerance test normal and STZ-induced diabetic rats	Decreasing in plasma glucose levels at 31.6 %.	[62, 63]
	Roots	Aqueous extract		*α*-Amylase inhibition	IC_50_ = 730.15 ± 0.25 *µ*g/mL	[63]
				*α*-Glucosidase inhibition	IC_50_ = 94.81 ± 5.99 *µ*g/mL	

*Eucalyptus globulus*	Leaves	Aqueous extract	150 and 300 mg/kg body weight intraperitoneally	Single and repeated oral STZ-induced diabetic rats	Exhibited a significant, dose-dependent hypoglycemic effect in streptozotocin-induced diabetic rats. The extract significantly increased the basal plasma insulin concentrations	[74]

*Nigella sativa*	Seeds	Aqueous extract	2 g/kg/day orally	Short-circuit current technique oral glucose tolerance test	Inhibited the electrogenic intestinal absorption of glucose *in vitro* chronic treatment improved glucose tolerance in rats also reduced body weight without any toxic effect.	[75]
	Seeds	Petroleum ether extract	2 g/kg/day during four-week intragastric gavage	STZ-induced diabetic rats	The petroleum ether extract exerts an insulin-sensitizing action by enhancing the activity of the two major intracellular signal transduction pathways of the hormone's receptor.	[76]
	Seeds	Ethanol extract	2 g/kg/day during four-week intragastric gavage	Oral glucose tolerance test on *Meriones shawi* rats	Hypoglycemic and hypolipidemic activity	[77]

*Calendula arvensis*	Flowers	Aqueous extract		*α*-Amylase	1368.27 ± 9.14 *µ*g/mL	[78]
				*α*-glucosidase	1121.10 ± 6.42 *µ*g/mL	
				*β*-Galactosidase	2116.82 ± 17.57 *µ*g/mL	
		Methanol extract		*α*-Amylase	573.37 ± 36.85 *µ*g/mL	
				*α*-Glucosidase	848.83 ± 49.93 *µ*g/mL	
		Hexane extract		*β*-Galactosidase	1422.66 ± 260.87 *µ*g/mL	
				*α*-Amylase	1955 ± 28.13 *µ*g/mL	
				*α*-Glucosidase	1722.59 ± 22.42 *µ*g/mL	
				*β*-Galactosidase	3156.98 ± 58.17 *µ*g/mL	

*Ziziphus Lotus*	Roots	Catechin		*α*-Glucosidase inhibition	IC_50_ = 87.55 ± 2.23 *µ*g/mL	[79]
	Fruits	Methanolic extract		*α*-Amylase inhibition	IC_50_ = 31.91 ± 1.53 *µ*g/mL	
	Leaves	Methanolic extract		*α*-Glucosidase inhibition	IC_50_ = 27.95 ± 2.45 *µ*g/mL	
				Α-Amylase inhibition	IC_50_ = 20.40 ± 1.30 *µ*g/mL	
				*α*-Glucosidase inhibition	IC_50_ = 8.66 ± 0.62 *µ*g/mL	

*Urtica dioica*	Aerial part	Aqueous extract	250 mg/kg	Oral glucose tolerance test (OGTT) at dose 250 mg/kg in normal and alloxan-induced diabetic rats.	Strong glucose-lowering effect in normal rats and no effect in alloxan-induced diabetic rats	[80]
*Urtica dioica*	Aerial part	Aqueous extract	Intraperitoneally 400 mg/L in the chronic treatment of streptozotocin-induced diabetic rats. 150 mg/kg in oral glucose tolerance test	Oral glucose tolerance test STZ-induced diabetic rats	Decrease in plasma glucose levels at 31.6. Significant reduction of hyperglycemia in oral glucose tolerance.	[62]

## Data Availability

The data used to support the findings of this study are included within the article.

## Additional Points

*Highlights.* Medicinal plants used by traditional healers to treat diabetes in the Taza region of Morocco were reported. Lamiaceae, Asteraceae, and Fabaceae were the most represented families*Salvia officinalis* L., *Marrubium vulgare,* and *Ajuga iva* are the most frequently used medicinal plants against diabetes.
